# Testing the Effectiveness of an Animated Decision Aid to Improve Recruitment of Control Participants in a Case-Control Study: Web-Based Experiment

**DOI:** 10.2196/40015

**Published:** 2022-08-26

**Authors:** Sandro T Stoffel, Jing Hui Law, Robert Kerrison, Hannah R Brewer, James M Flanagan, Yasemin Hirst

**Affiliations:** 1 Department of Behavioural Science and Health University College London London United Kingdom; 2 Institute of Pharmaceutical Medicine University of Basel Basel Switzerland; 3 Wolfson Institute of Population Health Queen Mary University of London London United Kingdom; 4 School of Health Sciences University of Surrey Guildford United Kingdom; 5 Department of Surgery and Cancer Imperial College London London United Kingdom

**Keywords:** animation, research participation, online experiment, case-control, recruitment, decision, effectiveness, epidemiology, recruitment, online, experiment, volunteer, survey, willingness, data, health research, research

## Abstract

**Background:**

Participation in case-control studies is crucial in epidemiological research. The self-sampling bias, low response rate, and poor recruitment of population representative controls are often reported as limitations of case-control studies with limited strategies to improve participation. With greater use of web-based methods in health research, there is a further need to understand the effectiveness of different tools to enhance informed decision-making and willingness to take part in research.

**Objective:**

This study tests whether the inclusion of an animated decision aid in the recruitment page of a study website can increase participants’ intentions to volunteer as controls.

**Methods:**

A total of 1425 women were included in a web-based experiment and randomized to one of two experimental conditions: one in which they were exposed to a simulated website that included the animation (animation; n=693, 48.6%), and one in which they were exposed to the simulated website without the animation (control; n=732, 51.4%). The simulated website was adapted from a real website for a case-control study, which invites people to consider taking part in a study that investigates differences in purchasing behaviors between women with and without ovarian cancer and share their loyalty card data collected through 2 high street retailers with the researchers. After exposure to the experimental manipulation, participants were asked to state (1) their intention to take part in the case-control study, (2) whether they would be willing to share their loyalty card for research, and (3) their willingness to be redirected to the real website after completing the survey. Data were assessed using ordinal and binary logistic regression, reported in percentages (%), adjusted odds ratio (AOR), and 95% confidence intervals.

**Results:**

Including the animation in the simulated website did not increase intentions to participate in the study (AOR 1.09; 95% CI 0.88-1.35) or willingness to visit the real study website after the survey (control 50.5% vs animation 52.6%, AOR 1.08; 95% CI 0.85-1.37). The animation, however, increased the participants’ intentions to share the data from their loyalty cards for research in general (control 17.9% vs animation 26%; AOR 1.64; 95% CI 1.23-2.18).

**Conclusions:**

While the results of this study indicate that the animated decision aid did not lead to greater intention to take part in our web-based case-control study, they show that they can be effective in increasing people’s willingness to share sensitive data for health research.

## Introduction

One of the most effective methods to test for exposure in epidemiological research is to conduct a case-control study in which people who have an illness are compared retrospectively with a matching population without the outcome [1]. Although it is a reliable methodology to test for associations, poor recruitment of population representative controls often undermines such studies [2].

Previous research reports the most common methods of recruiting control participants for case-control studies as follows: door-to-door recruitment, postal invitation, and random digit dialing [1,2]. More recently, with greater access to the internet, many cohort studies have moved their participant management and recruitment online (using unique websites), providing new opportunities to recruit participants, potentially improving diversity and ease of data collection [3,4]. Despite several advantages, however, caution needs to be exercised with the opportunistic recruitment of participants to web-based studies. For example, a recent study reported less than 4% of participants who visited a study recruitment website, after clicking on a targeted social media advertisement campaign, went on to sign up to the research [5]. This indicates that while individuals may be forming some interest to take part in research studies by clicking on a recruitment advertisement, their intention does not always translate to survey completion after they land on the research website.

Evidence on the barriers and facilitators of web-based survey completion primarily relates to the completion of stand-alone web-based surveys, rather than the use of unique websites to recruit participants to case-control studies [6-11]. These studies suggest that individuals’ trust in the organization carrying out the research, whether they are early adopters of technology and high in literacy, and whether the research is in line with the individuals’ values and beliefs are positive predictors of individual participation. Recommendations to achieve better outcomes include clear communication of the research goals, transparency about how data will be used, and shorter survey length.

Clinical trials have attempted to address some of the above (eg, transparency about how data will be used), using audio-visual decision aids to supplement the process of obtaining informed consent [12]. Communicating information via these mediums (enabled through web-based recruitment strategies), have the potential to reduce the associated cognitive load, facilitate further engagement with the research aims, generate positive attitudes toward the targeted behavior, and subsequently motivate engagement in the behavior itself [13-16]. To our knowledge, the potential impact of animated decision aids on intentions to take part in a web-based case-control study has not previously been investigated. This study aims to measure the effectiveness of an animated decision aid as a supplementary tool on a simulated website of a case-control study to encourage participation.

## Methods

### Setting

This study comprised a randomized web-based experiment, which assessed the effectiveness of adding an animated decision aid to a simulated website. The simulated website the animation was designed for (or added to) was the recruitment website for the case-control study: Cancer Loyalty Card Study (CLOCS) [17].

### Cancer Loyalty Card Study

CLOCS is an observational case-control study that aims to investigate the self-care behaviors of patients with ovarian cancer prior to their cancer diagnosis. It seeks to do this by investigating differences in transactional data (such as medication purchasing) between women with and without ovarian cancer (the transactional data are collected through the loyalty cards of 2 UK-based high street retailers). Cases (ie, women with ovarian cancer) are recruited through participating National Health Service sites, while controls are recruited through the study website. Full details for CLOCS have previously been reported in the study protocol [17].

### Animated Decision Aid

A key challenge for CLOCS recruitment has been communicating the research aims clearly, and our previous research highlights that the public often needs further explanations for how individual transactional data can be used in health research [18]. To improve public understanding and engagement with the aims of CLOCS, an animated decision aid was jointly prepared by Science Animated Limited, 2 patient representatives, and the CLOCS research team prior to the initiation of this web-based experiment. It aimed to convey key facts on ovarian cancer, the potential contribution of CLOCS in informing earlier diagnosis, as well as how women in the general population can play a role to aid its efforts (based on the participant information sheet tailored and approved by a National Health Service Research Ethics Committee [19/NW/0427-SA1], included in Multimedia Appendix 1). However, it should be noted that the animation was designed as a supplement to the main study materials, not as a key participant communication material to prompt informed decision-making. The animation features English subtitles and is 123 seconds (2 minutes and 3 seconds) long [19].

### Procedure

The randomized web-based experiment was programmed in Survey Monkey. In July 2020, women who were eligible to take part in CLOCS as a control(ie, between the ages of 18 and 70, living in the United Kingdom, and without an ovarian cancer diagnosis) were recruited through a survey vendor (Dynata Limited). For those who were interested in taking part, information about the experiment, including a brief description of CLOCS, was presented and followed by the completion of the study consent form. If participants consented and were eligible, they were randomized (in a 1:1 ratio) to one of the following two experimental conditions: the simulated CLOCS website without the animation (control), or the simulated CLOCS website with the animation (animation) (Multimedia Appendix 2 and 3).

Once everyone viewed the simulated website, they were asked to complete the survey, where they were required to indicate their intention to take part in CLOCS using a 4-point Likert scale (definitely yes, probably yes, probably not, and definitely not) adapted from previous research [20-22]. The study participants were then asked to indicate their loyalty card use by selecting from a list of high street retailers’ loyalty cards and whether they would be willing to share their loyalty card data for research purposes. The latter question was adapted from research on willingness to share electronic health data, which uses 4 commonly used models of consent for the use of data [23].

In the next step, the study participants were asked about their educational level, annual household income, and health literacy. For the latter, the eHealth Literacy Scale was used. This scale assesses individuals’ retrieval and judgement of health information on the internet [24]. It consists of 8 items rated on a 5-point scale, ranging from 1 to 5, and has demonstrated considerable reliability and validity [24]. The participants’ scores across the 8 items on the scale were summed and calculated for a sample mean. Individual scores below and above (or equal to) this sample mean were defined as low and high health literacy, respectively [25,26].

The final survey item was included as a behavior *proxy*. The participants were asked whether they want to be redirected to the actual CLOCS website for more information on how to take part. Those who responded that they would like to visit the website were provided with a link to the CLOCS website at the final page of the survey [22]. The website opened in a new tab for participants who clicked on the link. No data were collected from the study participants for their direct participation in CLOCS associated with the experiment.

### Ethics Approval

Ethics approval for this study was granted by the UCL Research Ethics Committee (Project ID: 17823/001).

### Data Analysis

A pilot study has been conducted beforehand for the purpose of sample size calculations. Based on the findings from the initial sample of 359 participants, with a 10% difference in intention to take part (definitely yes or probably yes versus definitely no or probably no), we determined the number of participants needed to achieve 95% certainty and 80% power was 650 per trial arm. Data from participants in both the pilot and final sample were combined for analysis.

Sample characteristics were assessed using descriptive statistics (Table S1 in Multimedia Appendix 4). To aid interpretation, the participants’ income and educational levels were dichotomized in inferential analyses. For income, we used £30,000 (US $37,000) as the cut-off point, based on the average household income in the United Kingdom (reported by the Office for National Statistics, 2020) [27]. For education, we categorized participants into those with General Certificate of Secondary Education or A Levels and those with a university degree [20-22].

Differences in intentions to take part in CLOCS were assessed using univariate and multivariate ordinal logistic regression. Willingness to visit the actual website and willingness to share loyalty card data for research purposes, between groups, were assessed using univariate and multivariate binary logistic regressions. Odds ratios (ORs), 95% confidence intervals, and *P* values are presented in the results, with *P* values below .05 regarded as statistically significant. Participants who spent a short amount of time completing the survey (ie, *survey speeders*) were excluded from the analysis (Multimedia Appendices 5-8, based on the 50% cut-off points provided from previous research using median values) [28]. We report the analysis for the whole sample in Table S2 (Multimedia Appendix 9) and the distribution of the time spent on the survey before and after the exclusion of the *speeders* in Multimedia Appendices 5-8 [29]. Additional analyses for interaction between the intervention and health literacy led to null results and were not reported due to the unbalanced proportion of those with low health literacy and high literacy in the study population.

## Results

### Study Sample

**Figure 1:**
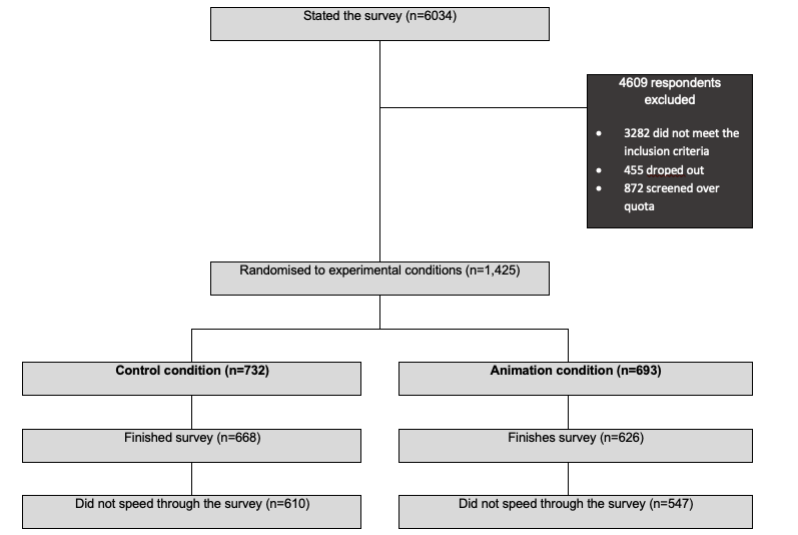
Flow through the study.

Figure 1 demonstrates the flow of participants through the study. In total, 6034 women were invited to participate, of which 4609 (76.4%) were excluded as they were not eligible, dropped out, or discontinued the initial screening. The remaining 1425 (23.6%) were then randomized to one of the following two experimental conditions: 732 (51.4%) were randomly allocated to the control condition, and 693 (48.6%) to the animation condition. Across conditions, 131 (9.2%) did not finish the survey after randomization. Furthermore, 137 (9.6%) were excluded as they spent less than 50% of the median time. The analytical sample consisted of 1157 women—610 (52.7%) participants in the control condition, and 547 (47.3%) in the animation condition.

Most women in the analytical sample were aged between 55 and 70 years (n=417, 36.9%), did not have a university degree (n=686, 59.3%), and had an annual household income of less than £30,000 (US $37,000; n=622, 53.8%). The mean eHealth Literacy Scale score for the participants was 30.3 out of 40; thus, those who scored below this mean were classified as having low health literacy [20,21]. Post hoc comparisons revealed that sociodemographic characteristics were comparable between the two experimental conditions (Table S1 in Multimedia Appendix 4).

### Intentions to Participate in CLOCS

**Figure 2:**
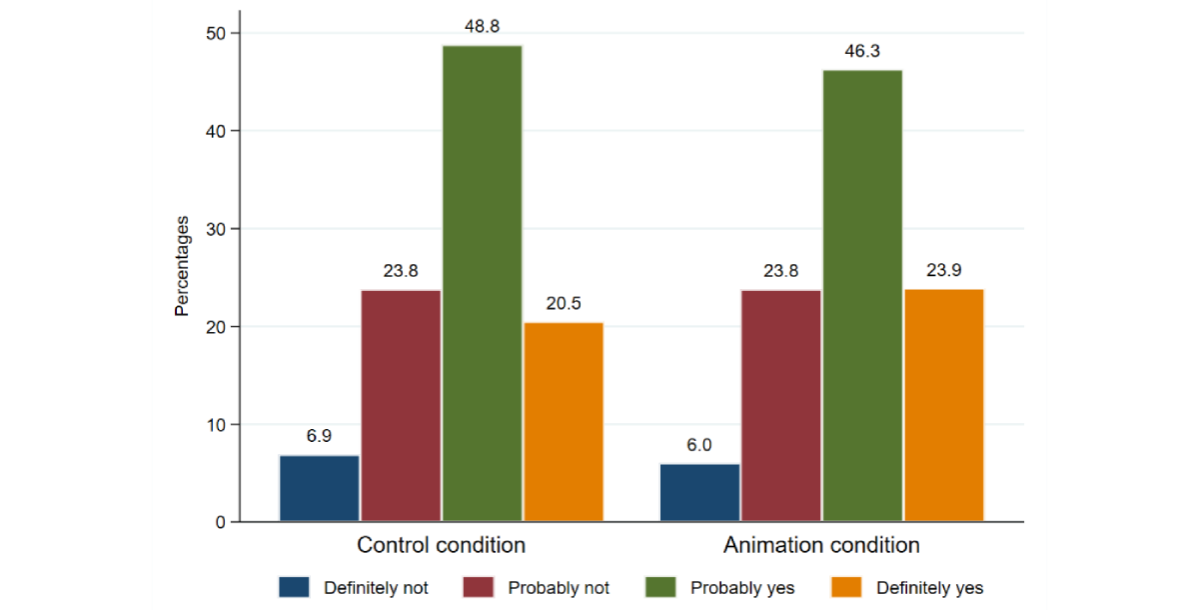
Distribution for intention to take part in Cancer Loyalty Card Study.

Intentions to participate in CLOCS were generally very high, with 69.7% (n=807) of women stating that they would probably or definitely participate. Figure 2 shows the distribution of the intentions to participate in CLOCS after exposure to the simulated website. The ordered logistic regressions in Table 1 show that the inclusion of the animation did not affect participation intentions (OR 1.12; 95% CI 0.90-1.39 and AOR 1.09; 95% CI 0.88-1.35). The regression further shows that older women aged 55-70 years stated lower intentions to participate (OR 0.56; 95% CI 0.42-0.74), while those with an income above average (OR 1.36; 95% CI 1.06-1.63), one or more existing loyalty cards (AOR 2.05; 95% CI 1.16-3.62), and low health literacy (AOR 1.26; 95% CI 1.01- 1.58) had higher intentions to take part in CLOCS.

**Table 1:** Ordered logistic regression on intention to participate in Cancer Loyalty Card Study (N=1157).

Variables		Unadjusted regression				Adjusted regression			
		AOR^a^	95% CI	*P* value	AOR		95% CI	*P* value	
**Condition**									
	Control	Reference	—^b^	—	Reference		—	—	
	Animation	1.120	0.904-1.389	.30	1.088		0.876-1.352	.45	
**Age (years)**									
	18-34	Reference	—	—	Reference		—	—	
	35-44	0.998	0.726-1.371	.99	0.952		0.691-1.311	.76	
	45-54	0.806	0.577-1.127	.21	0.799		0.569-1.122	.20	
	55-70	0.556	0.421-0.735	<.001	0.591		0.445-0.786	<.001	
**Education**									
	Below or equal to GCSE^c^	Reference	—	—	Reference		—	—	
	University degree	1.320	1.060-1.643	.01	1.145		0.909-1.441	.25	
**Income**									
	Below average	Reference	—	—	Reference		—	—	
	Above average	1.362	1.097-1.690	.01	1.263		1.006-1.584	.04	
**Card**									
	No	Reference	—	—	Reference		—	—	
	Yes	2.114	1.202-3.719	.009	2.051		1.164-3.616	.01	
**Health literacy**									
	High literacy	Reference	—	—	Reference		—	—	
	Low literacy	1.501	1.125-2.002	.006	1.381		1.029-1.854	.03	

^a^AOR: adjusted odds ratio.

^b^Not applicable.

^c^GCSE: General Certificate of Secondary Education.

### Willingness to Share Loyalty Card Data for Research

**Figure 3:**
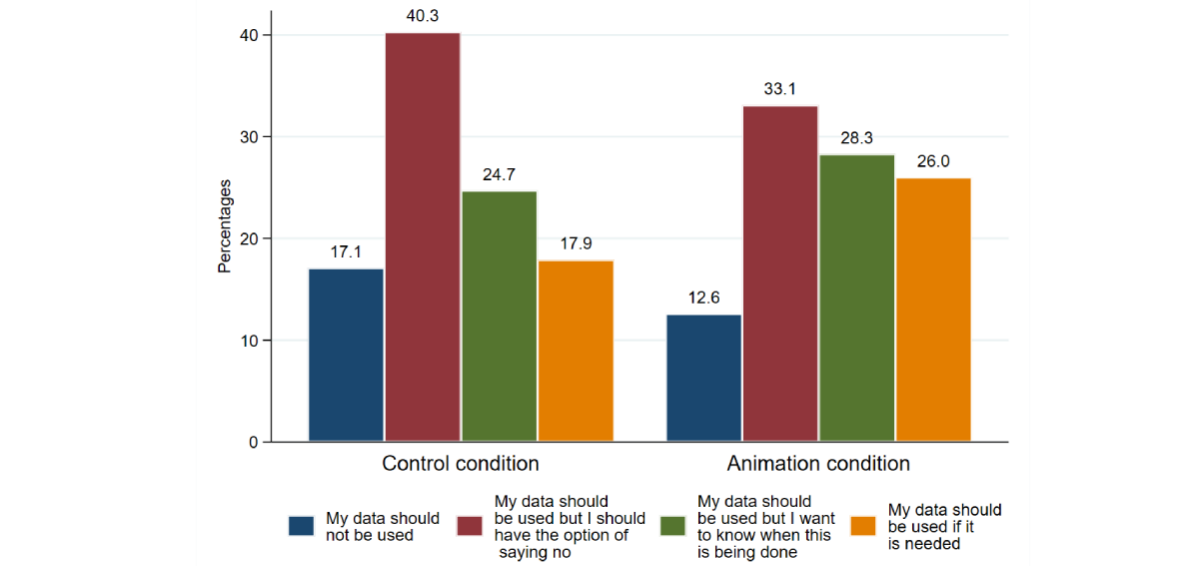
Distribution for willingness to share loyalty card data .

Most study participants stated that their data should be used but that they should have the option of saying no (control condition: n=246, 40.3%; and animation condition: n=181, 33.1%; Figure 3). However, significantly more participants in the animation condition, compared to control, indicated that they would be willing to provide the data if they were needed—as shown in the binary logistic regressions in Table 2 (control: n=109, 17.9% vs animation: n=142, 26.0%; OR 1.61; 95% CI 1.22-2.14 and AOR 1.64; 95% CI 1.23-2.18). Similarly, as with the intentions to participate in CLOCS, women aged 55-70 years were again less likely to state that their data should be used if needed compared to those aged 18-34 years (n=71, 17% vs n=72, 24.8%; AOR 0.62; 95% CI 0.43-0.89).

**Table 2:** Binary logistic regression on agreeing to share data from loyalty cards when needed (N=1157).

Variables		Total, n (%)	Unadjusted regression				Adjusted regression		
			AOR^a^	95% CI	*P* value	AOR		95% CI	*P* value
Overall		251 (21.7)	—^b^	—	—	—		—	—
**Condition**									
	Control	109 (17.9)	Reference	—	—	Reference		—	—
	Animation	142 (26.0)	1.612	1.216-2.136	.001	1.640		1.232-2.184	.001
**Age (years)**									
	18-34	72 (24.8)	Reference	—	—	Reference		—	—
	35-44	55 (22.9)	0.900	0.602-1.346	.61	0.864		0.575-1.299	.48
	45-54	53 (25.2)	1.022	0.678-1.540	.92	1.046		0.688-1.591	.83
	55-70	71 (17.0)	0.621	0.430-0.899	.01	0.601		0.411-0.878	.008
**Education**									
	Below or equal to GCSE^c^	147 (21.4)	Reference	—	—	Reference		—	—
	University degree	104 (22.1)	1.039	0.782-1.380	.79	0.932		0.690-1.260	.65
**Income**									
	Below average income	130 20.9)	Reference	—	—	Reference		—	—
	Above average income	121 (22.6)	1.106	0.836-1.464	.48	1.052		0.782-1.414	.74
**Card**									
	No	9 (20.9)	Reference	—	—	Reference		—	—
	Yes	242 (21.7)	1.048	0.496-2.216	.90	1.094		0.511-2.344	.82
**Health literacy**									
	High literacy	214 (22.0)	Reference	—	—	Reference		—	—
	Low literacy	37 (20.0)	0.886	0.599-1.309	.54	0.807		0.541-1.204	.30

^a^AOR: adjusted odds ratio.

^b^Not applicable.

^c^GCSE: General Certificate of Secondary Education.

### Willingness to Visit the CLOCS Website After the Survey

A slight majority of the study participants (n=596, 51.5%) indicated that they would like to visit the CLOCS website after the end of the survey (Multimedia Appendix 10). Table 3 shows that there was no difference between the two experimental conditions (control: n=308, 50.5% vs animation: n=288, 52.6%; OR 1.09; 95% CI 0.87-1.37 and AOR 1.08; 95% CI 0.85-1.37). Women with low health literacy, in comparison to those with high health literacy (n=111, 60% vs n=485, 49.9%; AOR 1.43; 95% CI 1.03-1.98), and women aged 35-44 years, in comparison to those aged 18-34 years, were more interested in visiting the study website (n=145, 60.4% vs n=148, 51%; AOR 1.49; 95% CI 1.05-2.12).

**Table 3:** Binary logistic regression on willingness to visit website after the survey (N=1157).

Variables		Total, n (%)	Unadjusted regression				Adjusted regression		
			AOR^a^	95% CI	*P* value	AOR		95% CI	*P* value
Overall		596 (51.5)	—^b^	—	—	—		—	—
**Condition**									
	Control	308 (50.5)	Reference	—	—	Reference		—	—
	Animation	288 (52.7)	1.090	0.865-1.374	.46	1.079		0.853-1.367	.53
**Age (years)**									
	18-34 years	148 (51.0)	Reference	—	—	Reference		—	—
	35-44 years	145 (60.4)	1.464	1.036-2.071	.03	1.489		1.048-2.115	.03
	45-54 years	118 (56.2)	1.231	0.861-1.758	.25	1.303		0.905-1.877	.16
	55-70 years	185 (44.4)	0.765	0.566-1.033	.08	0.825		0.606-1.123	.22
**Education**									
	Below or equal to GCSE^c^	337 (49.1)	Reference	—	—	Reference		—	—
	University degree	259 (55.0)	1.265	1.000-1.601	.05	1.214		0.946-1.559	.13
**Income**									
	Below average income	322 (51.8)	Reference	—	—	Reference		—	—
	Above average income	274 (51.2)	0.978	0.776-1.233	.85	0.865		0.677-1.107	.25
**Card**									
	No	18 (41.9)	Reference	—	—	Reference		—	—
	Yes	578 (51.9)	1.498	0.808-2.776	.20	1.521		0.810-2.854	.19
**Health literacy**									
	High literacy	485 (49.9)	Reference	—	—	Reference		—	—
	Low literacy	111 (60.0)	1.506	1.094-2.074	.01	1.431		1.031-1.986	.03

^a^AOR: adjusted odds ratio.

^b^Not applicable.

^c^GCSE: General Certificate of Secondary Education.

## Discussion

### Key Findings

This randomized web-based experiment examined the effectiveness of an animated decision aid to increase the willingness to participate in a case-control study. The results show that the animation did not increase intentions to participate in a real-world case-control study (CLOCS), or willingness to visit the real study website after the survey. However, the animation increased the participants’ willingness to share data from their loyalty cards for research. Interestingly, immediately after the completion of this web-based experiment, there was a spike in activity within the case-control study, with over 100 people signing up to participate in CLOCS.

### Comparison With Previous Literature

Our findings reflect the mixed evidence currently available in the literature. While there has been some support for the effectiveness of animated decision aids in the context of health behavior research, many studies have focused on how the interventions can improve participants’ knowledge of the health behavior concerned, as their main outcome. When assessing participant intention to engage in the behavior (or an objective measurement of the behavior itself), the findings have been more inconsistent [30,31].

An interesting finding in our study is that, among all participants, those with lower health literacy scores were more interested to find out about the study compared with those with high health literacy scores. Our sample size calculations were based solely on the primary outcome; thus, we might have been underpowered to detect the interaction effects of health literacy and outcomes on the behavior *proxy*. However, the previous studies indicate that multimedia interventions are not always significant in individuals with lower educational levels [14] and low health literacy [15,16]. It has been previously shown in the cancer screening literature that gist-based supplementary materials could be used to enhance engagement with the main literature among people with low numeracy [32], and perhaps using animation and other easy-to-read materials could enhance participant recruitment in health research [33]. Furthermore, the positive association between low health literacy and willingness to visit the website may be explained by factors such as wanting to find more information, the salience of the research topic, and other factors that were not included in this web-based experiment. As such, future studies focusing on the comprehension of the materials among people with low health literacy using a think-aloud methodology could explain this outcome.

### Strengths and Limitations

This study has some important limitations, which call for follow-up research. First, we did not include a comprehension assessment to check if participants fully understood or watched the animation. Second, we did not measure attitudes toward the simulated website and CLOCS. A recent systematic review on participant comprehension and informed consent in health research further highlighted that while there are efforts to improve participation rates using various methods, there is a lack of assessment of participant readability, literacy, and standardization of recruitment methods in health research for informed consent procedures [33]. While all the participant-facing CLOCS research materials have been reviewed by patient and public representatives, further assessment of participant comprehension prior to the animation experiment would have strengthened our methodology. However, the rationale for the exclusion of these measures was based on the assumptions that people often form immediate decisions about whether something is relevant to them using heuristic decision-making before establishing deliberative decisions [34]. As such, by excluding cognitive measures in our assessment to minimize judgement and bias, we tried to capture individuals’ potential reactions to the website as close to their reaction in real life. In this context, further studies using eye-tracking experiments on the simulated website will be highly informative to build a better understanding of the interaction with the website and the contents [35].

On the other hand, exposure to the animation increased the intentions to share loyalty card data for research in general, but not for intentions to participate in the CLOCS study; this suggests that there are study-specific characteristics that did not appeal to individuals (eg, actively signing up to provide information) or that the study participants were not eligible. However, a recent study also shows that only half of the population is willing to share shopping data for health research, highlighting the differences in sociodemographic characteristics of people who are willing to share their data for health research [36]. The characteristics of the participants who are willing to take part in CLOCS in this study mirror the results of this age-stratified survey employed in England, with older women less willing to take part. While self-sampling bias will continue to be a concern of case-control studies based on the differences in characteristics of the people who are willing to take part, recruitment strategies could be stratified and tailored to engage different populations who are less willing to take part in health research based on this evidence and the validation of public acceptability. Our results support this evidence further using experimental design with greater internal validity for potential barriers in recruiting participants to a case-control study.

### Implications for Policy and Future Research

While such questions of generalizability are warranted, this study still poses important relevance and implications to current research contexts. Due to the COVID-19 pandemic, many research studies have been forced to consider the possibility of being adapted online. This may require researchers to derive additional strategies to reach web-based samples with different characteristics. Underrepresentation in health research is already an issue for minority populations, people with low literacy, and those with greater deprivation using traditional methods of recruitment, unless they are specifically targeted [37,38]; thus, there is a further need to ensure that web-based strategies can provide means for researchers to attract representative samples.

Future studies should therefore continue exploring web-based methods to facilitate complex decision-making processes for potential participants of health research. The CLOCS animation has not been actively disseminated for participant recruitment following this dearth of evidence; however, unique findings might be obtained for research of a different nature. Other multimedia formats or mediums such as social media can be further explored in future studies, along with the consideration of potentially important variables such as participants’ willingness to share data for research purposes.

### Conclusion

The results of this study indicate that the animated decision aid did not influence the participants’ intention to take part in CLOCS or visit the study website. The animation, however, increased the probability of individuals stating that they would share their loyalty card data for research. Future research should continue exploring methods that can effectively engage participants with low health literacy to participate in complex health research.

## Appendix

Multimedia Appendix 1 Cancer Loyalty Card Study participant information sheet - health volunteer version 2.

Multimedia Appendix 2 A screenshot for the simulated website without the animation.

Multimedia Appendix 3 A screenshot of the simulated website with the animation.

Multimedia Appendix 4 Sample characteristics.

Multimedia Appendix 5 Distribution for time spent on the survey without excluding speeders (control group).

Multimedia Appendix 6 Distribution for time spent on the survey after excluding speeders (Control group).

Multimedia Appendix 7 Distribution for time spent on the survey without excluding speeders (Animation group).

Multimedia Appendix 8 Distribution for time spent on the survey excluding speeders (Animation group).

Multimedia Appendix 9 Adjusted regression models on the whole sample without excluding the speeders.

Multimedia Appendix 10 Distribution of willingness to visit the website after the survey.

## Abbreviations

AOR: adjusted odds ratio
CLOCS: Cancer Loyalty Card Study
OR: odds ratio
