# Supplementary material for: Testing the Effectiveness of an Animated Decision Aid to Improve Recruitment of Control Participants in a Case-Control Study: Web-Based Experiment
Source: J Med Internet Res. 2022 Aug 26;24(8):e40015. doi: 10.2196/40015 (PMC9463615; doi:10.2196/40015)
Supplement: Multimedia Appendix 1 [file jmir_v24i8e40015_app1.docx]

Participant Information Sheet – Healthy Volunteer

**Study title:** Cancer Loyalty Card Study (CLOCS)

- We would like to invite you to a research project that aims to investigate whether or not women have distinctive self-management behaviours prior to seeing their doctor with symptoms that are associated with ovarian cancer.
- Before you decide to take part, it is important that you understand why the research is being conducted and what it will involve. *Please take time to carefully read the following information and feel free to contact us if anything is not clear.*

**What is the purpose of the study?**

About 7,400 women are newly diagnosed with ovarian cancer in the UK every year, and 1 in 5 women diagnosed with ovarian cancer do not receive treatment, mostly because they’re too unwell to start treatment by the time they are diagnosed. This could partly be because ovarian cancer symptoms are vague and not well known. Women usually report in research that they did not think their symptoms were associated with ovarian cancer even when the symptoms became too painful and debilitating. We believe information about how often and what products women purchase (e.g. pain killers, digestive products and natural remedies) to self-manage these early symptoms could help identify ovarian cancer a few crucial weeks earlier.

The Cancer Loyalty Card Study (CLOCS) is focused on improving early detection of ovarian cancer.

We are investigating whether or not your everyday loyalty card use in high street retailers can help identify early signs of ovarian cancer. CLOCS aims to compare purchasing patterns of women with (cases) and without (controls) ovarian cancer diagnoses in order to potentially develop a way to detect cancer earlier.

**Who can take part in this study?**

Women 18 years and older who have not been diagnosed with ovarian cancer are eligible to take part in this study as a control participant. Ovarian cancer patients with loyalty cards at a participating high street retailer are eligible to take part in this study.

**What do I have to do?**

If you wish to take part after reading the information about the CLOCS, you will be able to complete the consent form and provide your loyalty card details on the website. Please also complete the short questionnaire and submit to the CLOCS team using our secure website. If the loyalty card details provided in the consent form do not match the high street retailer’s record and you have given consent for the CLOCS team to contact you again, we will contact you to clarify your loyalty card details. If you would like

to be informed of the progress and outcomes of this project, please visit the CLOCS website www.clocsproject.org.uk.

If you would prefer to complete the consent form and questionnaire on paper instead of the website, please return the consent form in this invitation in the free-post envelope, and we will send you the questionnaire in the post.

**What data will we be analysing? (Please also see the Participant Privacy Information Sheet)**

The first set of data will be collected by two high street retailers through your use of loyalty cards. This includes data about the items you purchased (e.g. paracetamol), when (i.e. date of purchase) and where you purchased them (i.e. store postcode). This will not include information on NHS prescriptions or any personally identifiable information, e.g. full name, postcode, ethnicity. Your loyalty card past purchase information will only be collected one time from the participating high street retailers. The CLOCS team will request your past purchase data from at most 7 years ago (depending on the high street retailer’s records and when you obtained a loyalty card) to the date you complete the consent form. ~~and questionnaire.~~

The second set of data will be collected through the participant questionnaire. Our questionnaire is designed specifically to investigate your ovarian cancer risk based on previous evidence. For this reason, we have included questions about you, your reproductive history, your cancer history (if any), other medical history, and about your loyalty card use.

Only the CLOCS research team will be able to access and analyse this data, and only the data needed for research purposes will be used, linked only by your unique barcode. You can request the list of information being used in this project from the research team via the contact information provided at the end of this information sheet.

**Will my taking part in the study be kept confidential? (Please also see the Participant Privacy Information Sheet)**

**YES.** Your identity will be kept confidential at all times. Only the research team will have access to the consent forms which will be kept in a secure locker and server. Once we receive your consent, we will share your consent for each specific retailer to request your data on your behalf. They will check your information to verify that the card details from your consent matches their data. If this does not match and you have consented for us to re-contact you, we will contact you requesting clarification of your card number. Otherwise, if we cannot contact you again, there will be no way to confirm your information, and we cannot receive your data. You will be given a unique study ID barcode which means all datasets will be pseudonymised, i.e. none of your identifiable information will be on our secure network.

You can change your consent to the study and your permission to the research team to access your past loyalty card data at any time by contacting us directly at [clocs@imperial.ac.uk](mailto:clocs@imperial.ac.uk) or contacting the principal investigator, Dr James Flanagan. Please do not share any sensitive information over email (e.g. your

date of birth).

The data will be analysed in a secure environment with access limited to the research team. No one other than the research team listed on this document will have access to the research dataset. All data will be stored and processed in the College’s ISO27001 certified secure environment – the “Secure Enclave”. This is a fully managed infrastructure and secure environment providing high availability, resilience and business continuity through multiple servers, back-ups and disaster recovery measures.

A robust data security model has been designed to protect sensitive personal and medical data from the potential risk of unauthorised access or distribution

We have strict data processing agreements in place with our commercial partners to ensure that your data is protected by us at all times. Your data will be transferred between the CLOCS team and the participating high street retailer using a secure encrypted file exchange. This means it will not be accessed by others. Your data will be mixed with similar participants without cancer so the commercial partners will not know your health status. Commercial partners will not have access to the study data and will not financially benefit from this project.

All outputs from this project will be made publicly available on our website and in academic platforms and will not include individual information. We will aim to publish outcomes in peer-reviewed academic journals and also on social media.

**What are the possible disadvantages and risks of taking part?**

There are no clear risks in taking part in this study**.** However, we understand that it may be emotional or distressing to think about cancer. If you are concerned about symptoms you are experiencing, please seek help and advice from health professionals. We intended to design the survey questions in the most sensible and sensitive way to ensure that there are no negative effects of this study on participants’ well-being. To our knowledge, this study is a unique project which could have important public health benefits for women. If the study is successful, this could be instrumental in raising cancer symptom awareness in commercial settings.

**What will happen to the results of the research study?**

The final study report will be produce summarising the information we have learned. If you are interested, please visit the CLOCS website for updates on the study’s progress and results. You can also register your participation on the website to stay updated on the CLOCS progress. www.clocsproject.org.uk

**What if something goes wrong?**

If you are harmed by taking part in this research project, there are no special compensation arrangements. If you are harmed due to someone's negligence, then you may have grounds for a legal action. Regardless of this, if you wish to complain, or have any concerns about any aspect of the way you have been treated

during the course of this study then you should immediately inform the Investigator Dr James Flanagan (j.flanagan@imperial.ac.uk).

The normal National Health Service mechanisms are also available to you such as contacting the local Patient Advice Liaison Services (PALS; pals@imperial.nhs.uk, 020 3313 0088). If you are still not satisfied with the response, you may contact the Imperial College, Joint Research Compliance Office.

**Who is organising and funding the research?**

The study is being organised by the CLOCS study team and sponsored by Imperial College London. This research is being funded by a Cancer Research UK Early Diagnosis Project Grant (C38463/A26726).

**Who has reviewed the study?**

IRAS Project ID: 262776

The study was first reviewed by patient representatives and edited accordingly. The pilot study was reviewed and received a favourable opinion from the University College London Ethics Committee and Consumer Data Research Centre (CDRC) at UCL, London. The project grant was reviewed by the Cancer Research UK Early Detection committee. This current study has also been reviewed by the NHS/HRA Research Ethics Committee (19/NW/0427).

**You can contact the following support lines for information on ovarian cancer and support:**

**Ovarian Cancer Action**

Call 0300 456 4700

**Macmillan Support Line**

Call 0808 808 00 00

**Cancer Research UK**

Call 0808 800 40 40

**We really appreciate your help. Thank you for taking the time to read this Participant Information Sheet.**

Sincerely,

Dr James Flanagan

*Principal Investigator*

*Ovarian Cancer Action Research Centre*

*Division of Cancer*

*Imperial College London*

If you have any questions arising from the Information Sheet or explanation already given to you, please ask the researchers on: [clocs@imperial.ac.uk](mailto:clocs@imperial.ac.uk)

This study has been reviewed and received favourable opinion from the _ Ethics Committee

Version 2_08 August 2019
