# Supplementary figures and images for: Testing the Effectiveness of an Animated Decision Aid to Improve Recruitment of Control Participants in a Case-Control Study: Web-Based Experiment
Source: J Med Internet Res. 2022 Aug 26;24(8):e40015. doi: 10.2196/40015 (PMC9463615; doi:10.2196/40015)

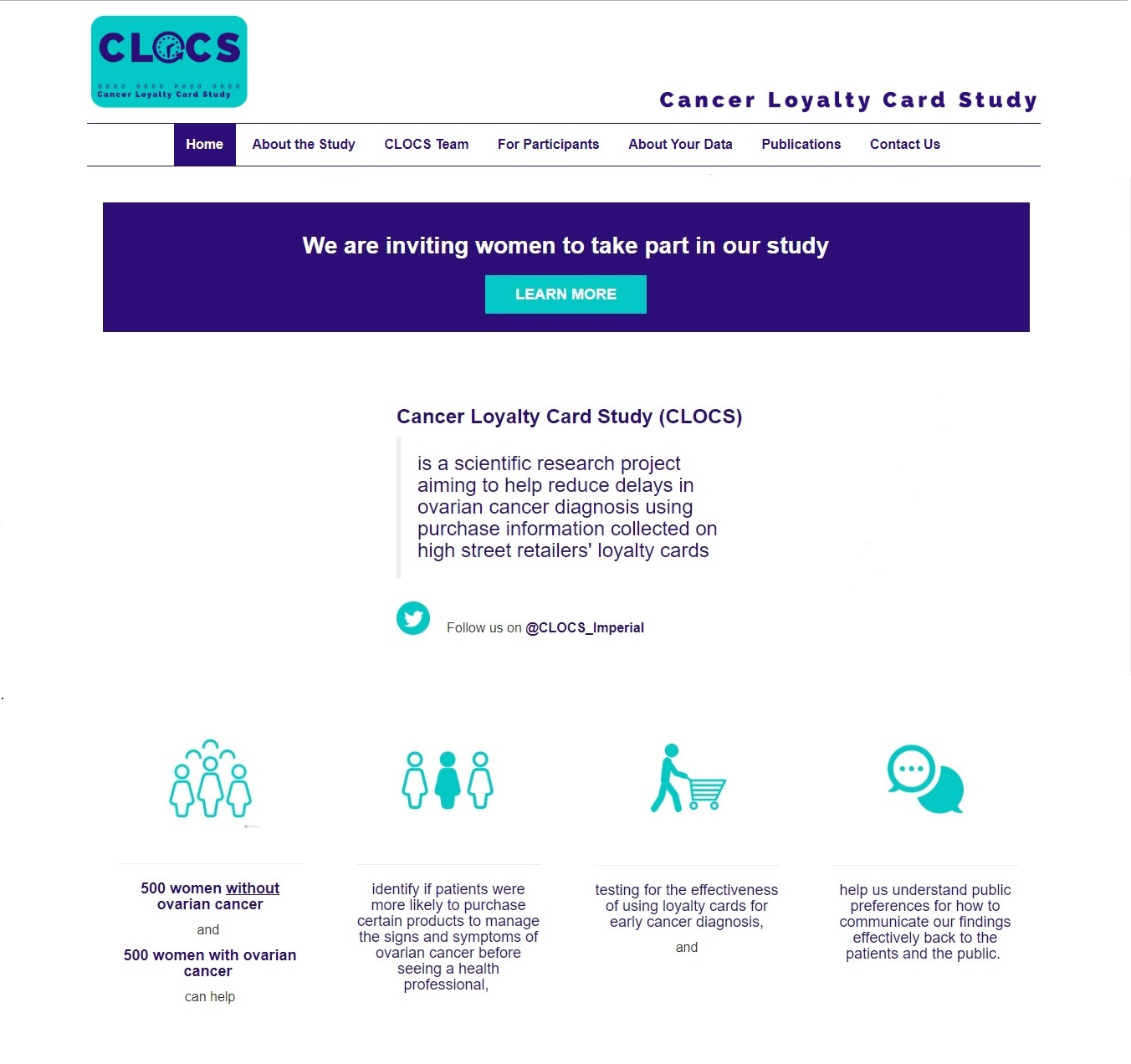

Supplement: Multimedia Appendix 2 [file jmir_v24i8e40015_app2.png]

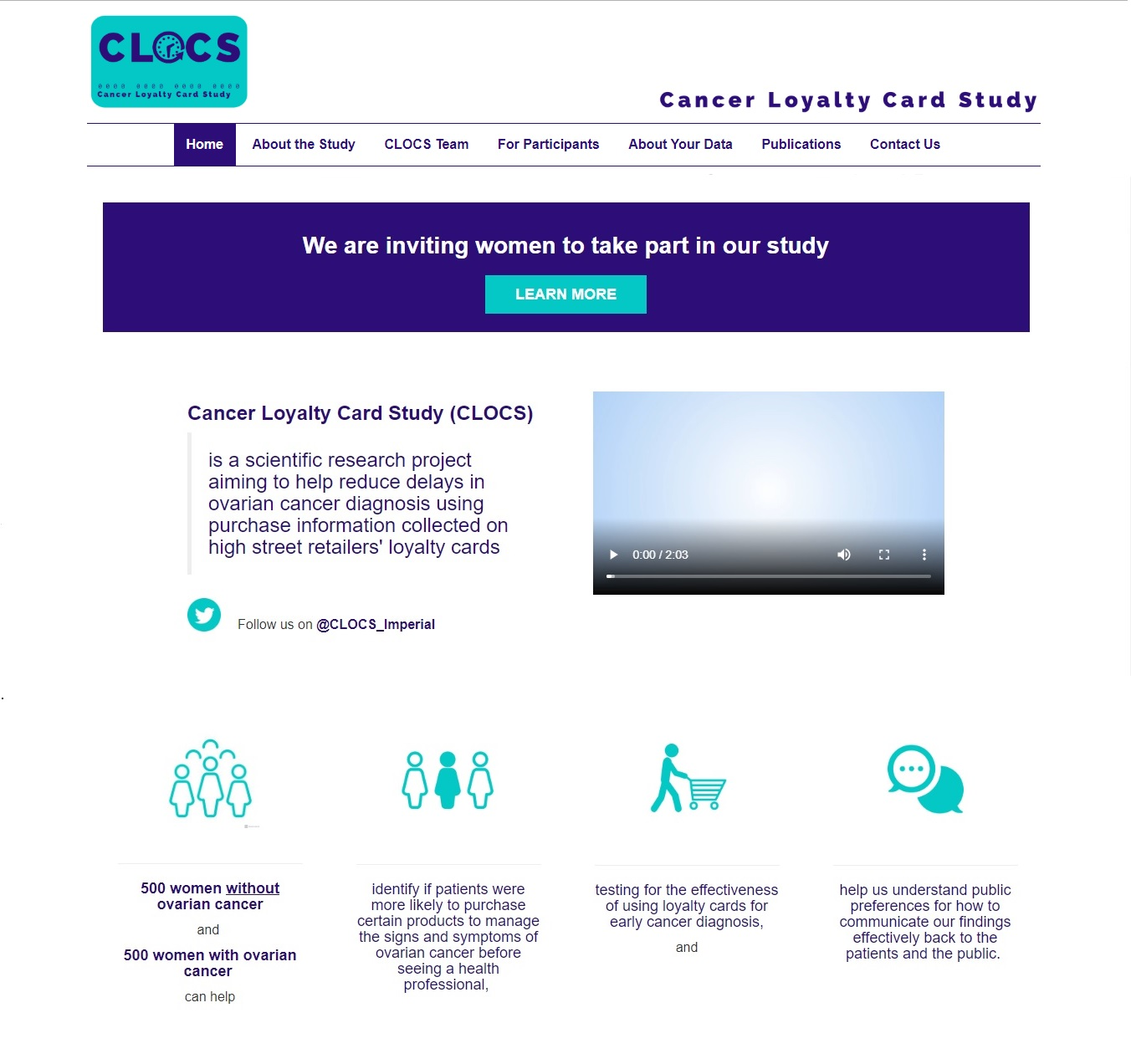

Supplement: Multimedia Appendix 3 [file jmir_v24i8e40015_app3.png]

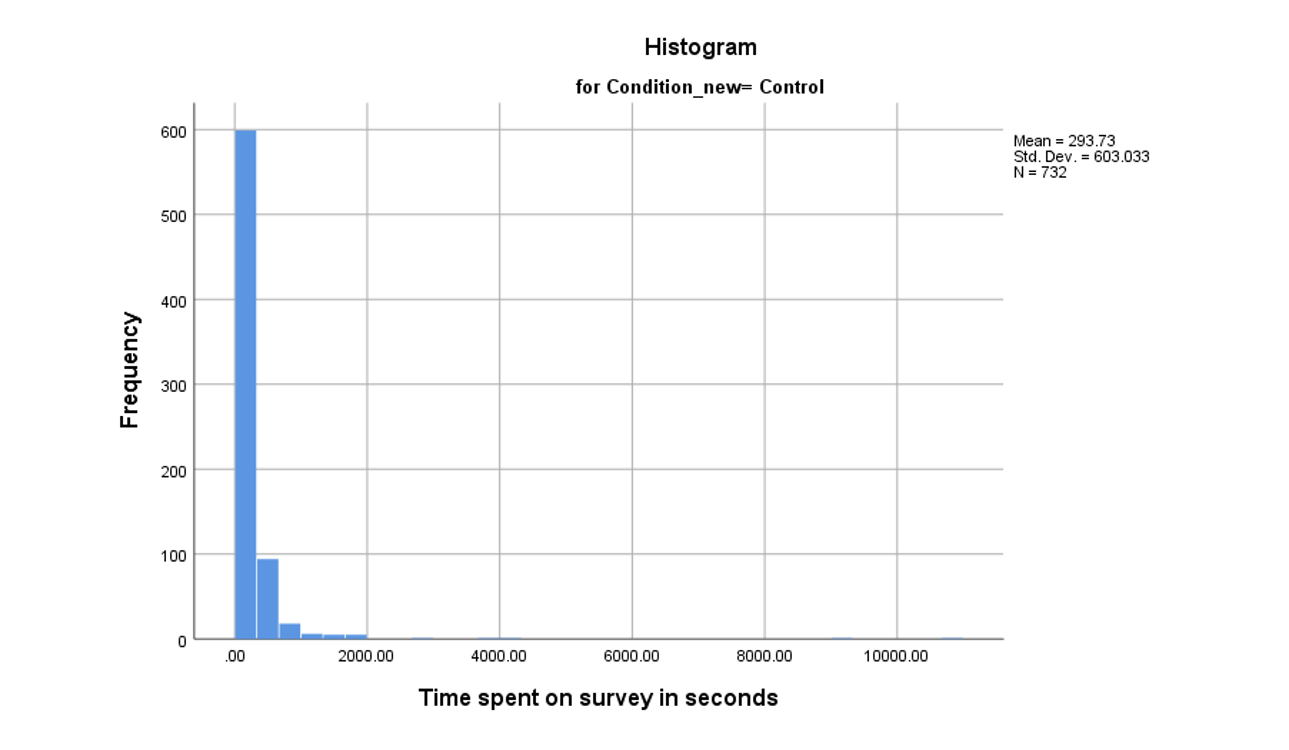

Supplement: Multimedia Appendix 5 [file jmir_v24i8e40015_app5.png]

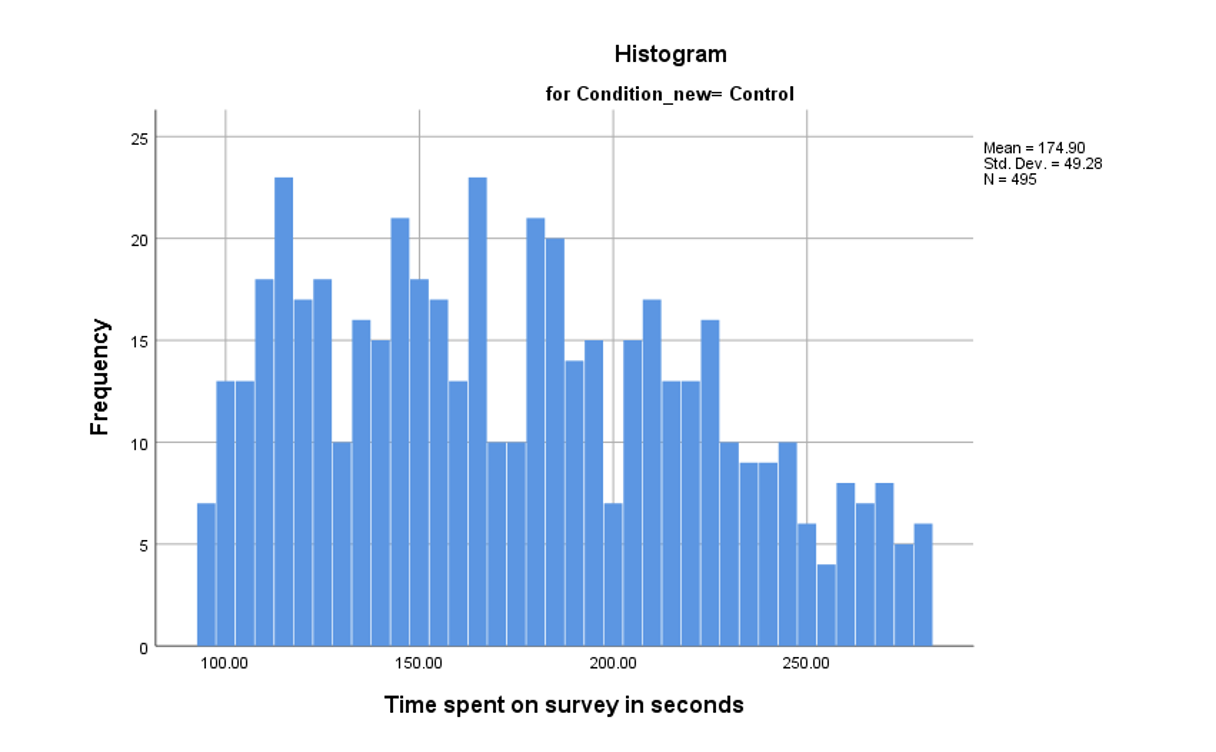

Supplement: Multimedia Appendix 6 [file jmir_v24i8e40015_app6.png]

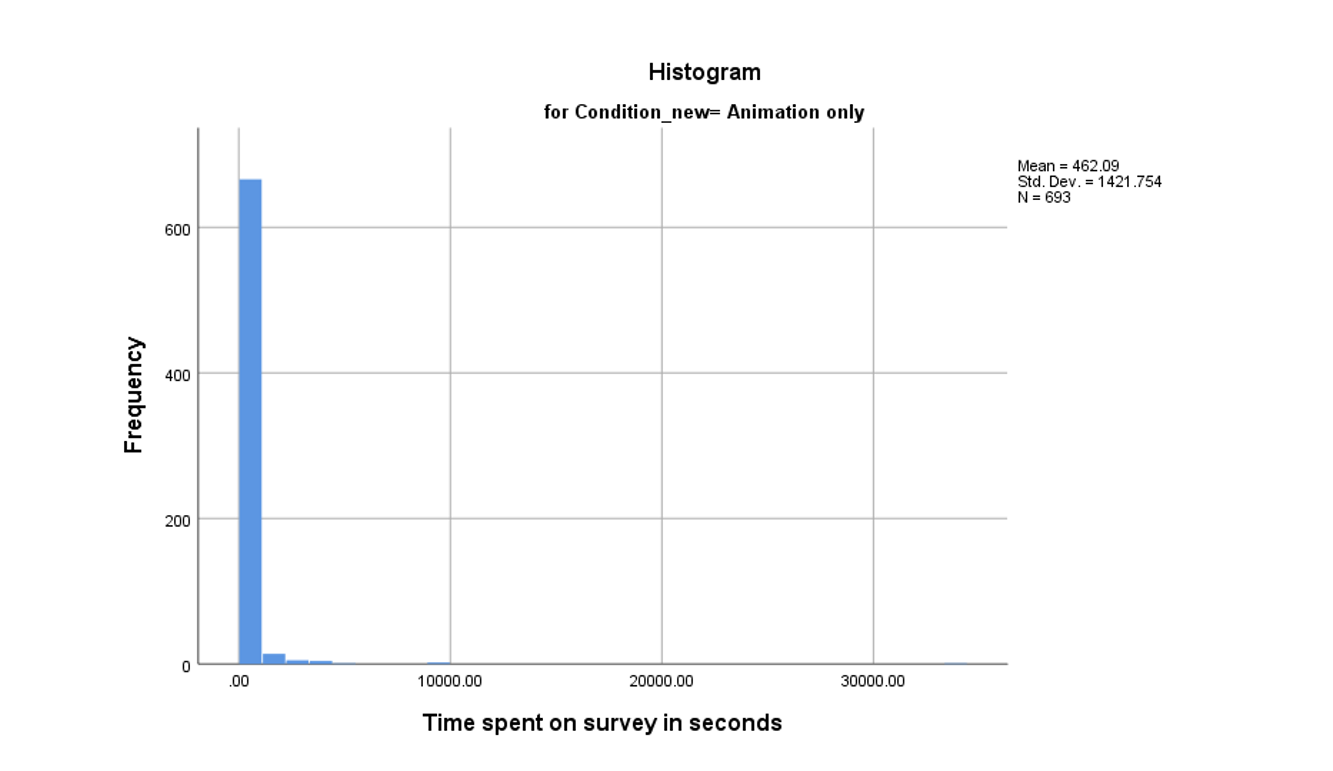

Supplement: Multimedia Appendix 7 [file jmir_v24i8e40015_app7.png]

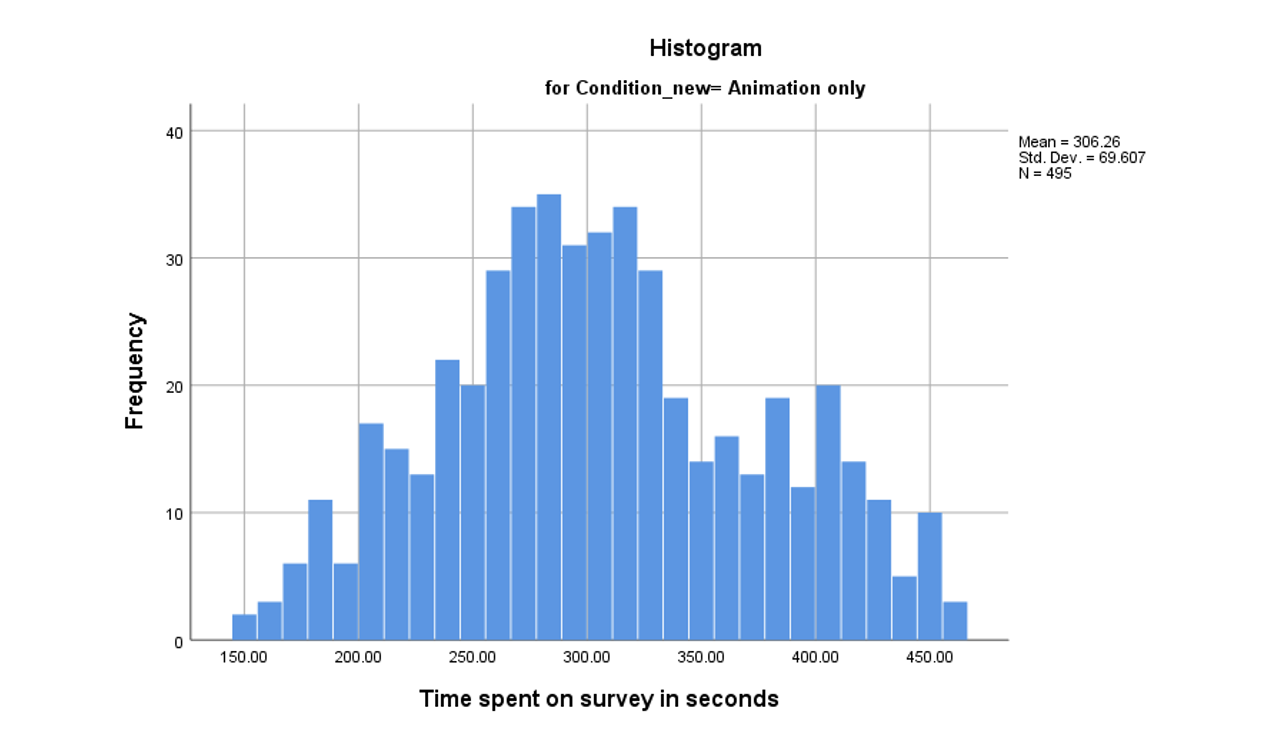

Supplement: Multimedia Appendix 8 [file jmir_v24i8e40015_app8.png]

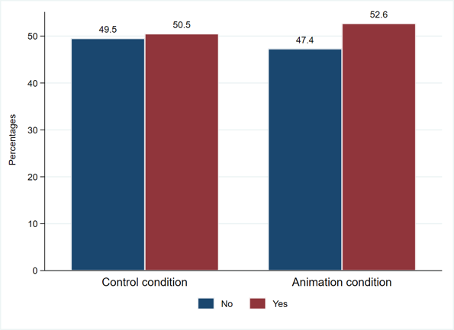

Supplement: Multimedia Appendix 10 [file jmir_v24i8e40015_app10.png]
