# Supplementary material for: Testing the Effectiveness of an Animated Decision Aid to Improve Recruitment of Control Participants in a Case-Control Study: Web-Based Experiment
Source: J Med Internet Res. 2022 Aug 26;24(8):e40015. doi: 10.2196/40015 (PMC9463615; doi:10.2196/40015)
Supplement: Multimedia Appendix 4 [file jmir_v24i8e40015_app4.docx]

Table S1. Socio-demographic characteristics of the study sample

|  | Control condition (N=610) | | Animation condition (N=547) | | Total (N=1,157) | |
| --- | --- | --- | --- | --- | --- | --- |
| Variable | N | (%) | N | (%) | N | (%) |
| Age |  |  |  |  |  | |
| 18-34 years | 147 | (24.1) | 143 | (26.1) | 290 | (25.1) |
| 35-44 years | 108 | (17.7) | 132 | (24.1) | 240 | (20.7) |
| 45-54 years | 131 | (21.5) | 79 | (14.4) | 210 | (18.5) |
| 55-70 years | 224 | (36.7) | 193 | (35.3) | 417 | (36.0) |
| Education |  |  |  |  |  |  |
| Below or equal to GCSEs | 379 | (62.1) | 307 | (56.1) | 686 | (59.3) |
| University degree | 231 | (37.9) | 240 | (43.9) | 471 | (40.7) |
| Income |  |  |  |  |  |  |
| Below average income | 341 | (55.9) | 281 | (51.4) | 622 | (53.8) |
| Above average income | 269 | (44.1) | 266 | (48.6) | 535 | (46.2) |
| Card |  |  |  |  |  |  |
| No | 18 | (3.0) | 25 | (4.6) | 43 | (3.7) |
| Yes | 592 | (97.0) | 522 | (95.4) | 1,114 | (96.3) |
| Health literacy |  |  |  |  |  |  |
| High literacy | 516 | (84.6) | 456 | (83.4) | 972 | (84.0) |
| Low literacy | 94 | (15.4) | 91 | (16.6) | 185 | (16.0) |
