# Supplementary material for: Testing the Effectiveness of an Animated Decision Aid to Improve Recruitment of Control Participants in a Case-Control Study: Web-Based Experiment
Source: J Med Internet Res. 2022 Aug 26;24(8):e40015. doi: 10.2196/40015 (PMC9463615; doi:10.2196/40015)
Supplement: Multimedia Appendix 9 [file jmir_v24i8e40015_app9.docx]

Table S2. Adjusted regression models on the whole sample without excluding speeders

|  | Intention to participate in study | | Intention to share data | | Willingness to visit website | |
| --- | --- | --- | --- | --- | --- | --- |
| Variable | aOR | 95% CI | aOR | 95% CI | aOR | 95% CI |
| Condition |  |  |  |  |  |  |
| Control | Ref. |  | Ref. |  | Ref. |  |
| Animation | 0.968 | 0.789 - 1.188 | 1.529 | 1.166 - 2.006** | 0.972 | 0.778 - 1.213 |
| Age |  |  |  |  |  |  |
| 18-34 years | Ref. |  | Ref. |  | Ref. |  |
| 35-44 years | 0.998 | 0.745 - 1.338 | 0.837 | 0.573 - 1.221 | 1.324 | 0.963 - 1.820 |
| 45-54 years | 0.890 | 0.647 - 1.225 | 1.125 | 0.759 - 1.668 | 1.162 | 0.826 - 1.635 |
| 55-70 years | 0.704 | 0.541 - 0.917** | 0.628 | 0.439 - 0.898* | 0.817 | 0.613 - 1.089 |
| Education |  |  |  |  |  |  |
| Below or equal to GCSEs | Ref. |  | Ref. |  | Ref. |  |
| University degree | 1.193 | 0.959 - 1.484 | 1.000 | 0.751 - 1.330 | 1.216 | 0.960 - 1.540 |
| Income |  |  |  |  |  |  |
| Below average income | Ref. |  | Ref. |  | Ref. |  |
| Above average income | 1.214 | 0.979 - 1.505 | 1.081 | 0.816 - 1.433 | 0.852 | 0.675 - 1.074 |
| Card |  |  |  |  |  |  |
| No | Ref. |  | Ref. |  | Ref. |  |
| Yes | 2.180 | 1.267 - 3.750** | 1.028 | 0.499 - 2.118 | 1.566 | 0.858 - 2.860 |
| Health literacy |  |  |  |  |  |  |
| High literacy | Ref. |  | Ref. |  | Ref. |  |
| Low literacy | 1.293 | 0.990 - 1.687 | 0.843 | 0.588 - 1.209 | 1.324 | 0.988 - 1.776 |
|  | 1,294 |  | 1,294 |  | 1,294 |  |

* *p*<0.05; ** *p*<0.01
